# Case report: Transcatheter edge-to-edge repair with MitraClip for acute mitral regurgitation after myocardial infarction

**DOI:** 10.1097/MD.0000000000036230

**Published:** 2023-12-01

**Authors:** Fan-Qi Meng, Bin Wang, Xiang Chen, Mao-Long Su, Peng-Long Wu, Yan Wang

**Affiliations:** a Department of Cardiology, Xiamen Cardiovascular Hospital of Xiamen University, School of Medicine, Xiamen University, Xiamen, Fujian Province, China; b Department of Echocardiography, Xiamen Cardiovascular Hospital of Xiamen University, School of Medicine, Xiamen University, Xiamen, Fujian Province, China.

**Keywords:** Cardiogenic shock, extracorporeal membranous oxygenation, MitraClip, myocardial infarction, papillary muscle rupture

## Abstract

**Introduction:**

Acute mitral regurgitation (MR) due to papillary muscle rupture (PMR) is a rare but lethal mechanical complication of acute myocardial infarction (MI). The treatment of patients with post-MI PMR, especially those with cardiogenic shock, presents great challenges due to the high surgical risk.

**Patient concerns:**

We report an 80-year-old woman with a history of hypertension and diabetes mellitus, presented with chest pain. Despite an early percutaneous coronary intervention and transfer to the intensive care unit, her general condition and hemodynamic parameters continued to deteriorate rapidly.

**Diagnosis:**

Evidenced by electrocardiogram, echocardiogram and coronary angiography, the patient was diagnosed with acute lateral and posterior ST-segment elevation MI, cardiogenic shock, PMR, severe MR, and pulmonary edema.

**Interventions:**

The patient received percutaneous mitral valve repair with MitraClip (Abbott Vascular, Santa Clara, CA, USA) supported by extracorporeal membranous oxygenation and intra-aortic balloon pump.

**Outcomes:**

The patient was discharged with relief of heart failure symptoms, reduced MR, and recovery of cardiac function, remaining in a stable condition in New York Heart Association class I after 15-month outpatient follow up.

**Conclusion:**

Transcatheter edge-to-edge repair with MitraClip can serve as a viable alternative to surgery in reducing MR in post-MI PMR patients at high surgical risk.

## 1. Introduction

Acute mitral regurgitation (MR) due to papillary muscle rupture (PMR) is a rare but lethal mechanical complication of acute myocardial infarction (MI). Although the incidence of PMR in patients with acute MI has declined to 0.05% to 0.26% in the reperfusion era, it continues to be associated with a high mortality rate of 10% to 40%.^[1]^ Surgical mitral valve replacement or repair (SMVR) remains the standard therapy, but only 58% of patients with post-MI PMR undergo SMVR due to the high surgical risk, including old age, comorbidities, and unstable hemodynamics.^[2]^ Percutaneous mitral valve repair (PMVR) using transcatheter edge-to-edge repair (TEER) with MitraClip has been confirmed as a promising alternative to surgery for reducing MR in high-risk patients^[3]^; however, there are limited reports of its use for post-MI PMR. Here, we report a patient with post-MI PMR and cardiogenic shock, who underwent extracorporeal membranous oxygenation (ECMO) support and successful MVR using TEER with the MitraClip device.

## 2. Case report

An 80-year-old woman presented with chest pain for 6 hours. She complained of acute precordial chest pain radiating to her arm and neck. Her chest pain began approximately 6 hours previously and had not completely resolved since its onset. Her medical history included hypertension and type 2 diabetes mellitus. The patient denied a family history of a specific genetic disease. On physical examination, the critical signs were as follows: body temperature 36.4°C, pulse 92 beats per min; respiratory rate 20 breaths per min, and blood pressure 75/43 mm Hg. Chest auscultation revealed bilateral cracks. Heart sounds were normal with regular rate and rhythm. There was a grade 4/6 systolic murmur at the cardiac apex. Extremities were cold with weak pulses. Cardiac injury biomarkers were notably elevated on admission: myoglobin 613 ng/mL, troponin T 492 ng/L, creatine kinase 219.8 U/L, and creatine kinase myocardial band 22.3 U/L. Troponin T was further increased to over 10,000 ng/L in 4 hours. Electrocardiography showed ST elevation of 0.2 to 0.4 mV in leads V5–V9, and ST depression of 0.1 to 0.4 mV in leads V1 to V4. Transthoracic echocardiography revealed severe eccentric MR due to PMR with a preserved left ventricular ejection fraction of 67%.

The final diagnosis was acute lateral and posterior ST-segment elevation MI, cardiogenic shock, PMR, severe MR, and pulmonary edema. The differential diagnoses included aortic dissection, pulmonary embolism, and fulminant myocarditis.

Emergency coronary angiography demonstrated 70% midsegment stenosis in the left anterior descending artery, 70% proximal stenosis, distal 100% occlusion in the left circumflex (LCx) artery, and normal right coronary artery. Percutaneous coronary intervention of the LCx artery was successfully performed with the assistance of an intra-aortic balloon pump (IABP).

The patient was transferred to the cardiac intensive care unit (CICU). Although the mean arterial pressure was maintained at 70 to 75 mm Hg with the support of IABP and low-dose dobutamine infusion at 3 to 5 µg/kg/min, pulmonary edema was exacerbated despite high doses of intravenous furosemide. On day 4, the patient was intubated and underwent continuous renal replacement therapy. Even though pulmonary congestion was relieved and oxygenation improved, the hemodynamics began to deteriorate. On day 6, venous-arterial-ECMO was initiated, which stabilized her hemodynamic parameters and improved organ perfusion.

**Figure 1.**
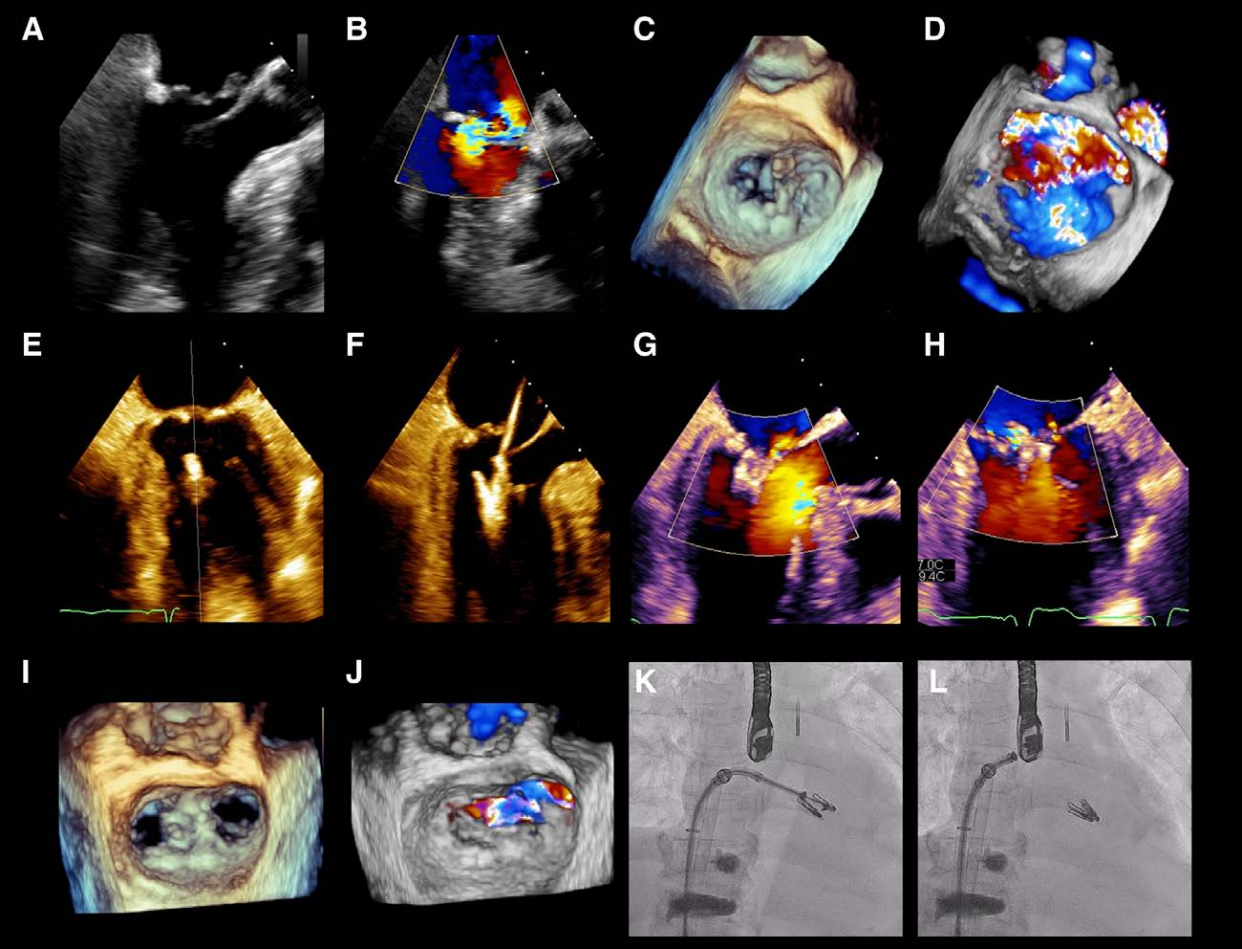
Echocardiography and fluoroscopic images of transcatheter edge-to-edge repair. (A and B) Transesophageal echocardiography (TEE): mid-esophageal long-axis view and color flow depicting severe eccentric mitral regurgitation (MR) due to prolapsed posterior leaflet. (C and D) 3D-TEE image and color flow demonstrating ruptured posteromedial papillary muscle and associated posterior (P2 scallop) leaflet flail with huge MR. (E and F) X-plane view demonstrating implantation of the first clip. (G and H) X-plane view with color flow demonstrating mild MR after implantation of 2 clips. (I and J) 3D-TEE image with surgical *en face* view of mitral valve depicting double orifice after implantation of 2 clips and mild MR. (K and L) Fluoroscopic image after implantation of 2 clips. 3D-TEE = three-dimensional transesophageal echocardiography.

Three-dimensional transesophageal echocardiography (3D-TEE) demonstrated posteromedial PMR, prolapsed P2 scallop of the posterior leaflet, and severe eccentric MR (4+) with effective regurgitant orifice area 0.75 cm^2^ (Fig. 1A–D; see Videos, Supplemental Video 1–4, http://links.lww.com/MD/K805, http://links.lww.com/MD/K806, http://links.lww.com/MD/K807, http://links.lww.com/MD/K808, which demonstrated ruptured papillary muscle and MR). Calcification of the posterior leaflets and the mitral valve annulus was also observed. Anterior and posterior leaflet lengths were respectively 21 and 10 mm. The flail width was 10.4 mm and the flail gap was 7.2 mm. The mitral valve area was 4.63 cm^2^ and the mean transmitral gradient was 4 mm Hg. The patient was adequately evaluated by a multidisciplinary heart team, which included a cardiac intensivist, cardiac surgeon, and interventional cardiologist, and was considered to be at high surgical risk, with a EuroSCORE II score of 33% and STS score of 38%. She was referred for emergency TEER with a MitraClip (Abbott Vascular, Santa Clara, CA, USA).

On day 7, after thorough preoperative preparation under general anesthesia, the clip surgery was conducted under fluoroscopic and 3D-TEE guidance in a hybrid laboratory. A 24F MitraClip steerable guide catheter was delivered into the left atrium via the left femoral venous route and trans-septal puncture. The clip delivery system was introduced into the left atrial and left ventricular surfaces of the mitral valve in sequence. A MitraClip XTR and NTR were successfully implanted at the A2 to P2 scallop (Fig. 1E–H; see Video, Supplemental Video 5, http://links.lww.com/MD/K809, which showed implantation of the first clip). 3D-TEE demonstrated a noticeable reduction of MR from 4 + to 1+, with a mean transmitral gradient of 2 to 4 mm Hg (Fig. 1I–L; see Video, Supplemental Video 6–9, http://links.lww.com/MD/K810, http://links.lww.com/MD/K811, http://links.lww.com/MD/K812, http://links.lww.com/MD/K813, which showed implantation of the second clip and mild MR). After the MitraClip procedure, the left atrial pressure dropped from 16/12/15 to 10/9/9 mm Hg. Due to rapid hemodynamic recovery, the patient was weaned off ECMO immediately after the procedure, and IABP was withdrawn the next day. Tracheal intubation was removed on postoperative day 4. She recovered uneventfully and left the CICU on postoperative day 12, and successful hospital discharge was eventually achieved. At the 7-month follow-up, echocardiography revealed moderate MR (MR2+), effective regurgitant orifice area 0.25 cm^2^, and a mean transmitral gradient of 5 mm Hg. Follow-up results at 15 months suggested a stable mitral valve clamping position, moderate MR2+, and an ejection fraction of 65%. She remains in a stable clinical condition in New York Heart Association class I.

## 3. Discussion

PMR, as a mechanical complication of MI, usually causes severe hemodynamic disturbances, requiring prompt recognition and urgent medical attention. As a consequence of reperfusion therapy, there has been a decline in the incidence of post-MI PMR to 0.05% to 0.26%.^[1]^ A study based on the National Inpatient Sample database (2003–2015) in the USA revealed that PMR occurred in 0.05% of ST-elevation myocardial infarctions and 0.01% of non-ST-elevation myocardial infarctions hospitalization, with no significant change in incidence over the 13 years in either cohort.^[2]^ However, the mortality of post-MI PMR remains high between 10% and 40%.^[1]^

PMR commonly occurs within 3 to 5 days of acute MI.^[1]^ The mitral valve is typically supported by 2 PMs, the anterolateral and posteromedial branches. Posteromedial PMR is observed ~3 times more often than rupture of the anterolateral branch.^[3]^ The anterolateral PM is less prone to rupture due to its dual blood supply from the left anterior descending and LCx arteries, whereas the posteromedial PM is more susceptible to ischemic injury due to its solitary blood supply from the posterior descending artery derived from the LCx or right coronary artery. PMR can be partial or complete, which may influence the severity of MR and clinical status. In this case, PMR was observed within 6 hours after the onset of ischemic chest pain, and the culprit artery was identified as the LCx. Echocardiography revealed posteromedial PMR, which contributed to the prolapsed posterior leaflet and massive MR. Due to severe eccentric MR, the patient presented with pulmonary edema that quickly progressed to cardiogenic shock.

Often, patients with post-MI PMR require active medical care in the CICU, including invasive mechanical ventilation, vasoactive medications, inotropes, diuretics, or ultrafiltration, reduction of the cardiac burden, and increased forward stroke volume. In the case of refractory cardiogenic shock, some form of short-term mechanical circulatory support, such as IABP, ECMO or a percutaneous ventricular assist device (Impella or Tandem Heart), should be applied as a bridge to the definitive surgical or percutaneous therapies.^[1]^ In our patient, an IABP was implanted upon admission to the CICU, and pulmonary edema improved with mechanical ventilation, diuretics, and continuous renal replacement therapy. However, it has become increasingly difficult to maintain arterial pressure due to decreased circulating blood volume and severe MR, and ECMO had to be implanted to support.

Even though SMVR remains the standard therapy for post-MI PMR, it is generally challenging to decide on surgery due to high surgical risk or unstable hemodynamics.^[4]^ PMVR with MitraClip has recently emerged as a promising alternative to severe MR. Recently a prospective, multicenter, randomized controlled study included 471 patients who had at least symptomatic MR grade 3 + within 90 days after MI and reported that the immediate procedural success rate did not differ between SMVR and PMVR (92% vs 93%, *P* = .53), while in-hospital and 1-year mortality rates were significantly higher in SMVR than in PMVR (16% vs 6%, *P* = .03 and 31% vs 17%, *P* = .04).^[5]^ However, patients with PMR were excluded from this study due to their worse and ambiguous prognosis.^[5]^ The use of PMVR in the setting of post-MI PMR has been reported in a few successful cases,^[6–9]^ in which surgical risks were typically prohibitive. Multidisciplinary team-based discussions have been advocated to determine the optimal approach for definitive therapy.^[1]^ In the present case, following the use of ECMO as a bridge to definitive therapy, TEER with MitraClip was chosen due to the enormous surgical risk (EuroSCORE II score of 33% and STS score of 38%). In this patient, despite the considerable challenges, the MitraClip procedure was accomplished, resulting in a dramatic reduction of regurgitation without a corresponding increase in the mean transmitral gradient. The medium-term follow-up showed favorable outcomes in this patient.

## 4. Conclusion

This report shows that ECMO may be lifesaving in post-MI PMR patients with cardiogenic shock, and TEER with MitraClip can provide a viable alternative to surgery in reducing MR in post-MI PMR patients at a high surgical risk.

## Author contributions

**Conceptualization:** Fanqi Meng, Yan Wang.

**Data curation:** Bin Wang, Xiang Chen, Peng-Long Wu.

**Formal analysis:** Mao-Long Su.

**Funding acquisition:** Fanqi Meng.

**Investigation:** Fanqi Meng, Xiang Chen.

**Methodology:** Bin Wang, Xiang Chen, Mao-Long Su.

**Project administration:** Yan Wang.

**Resources:** Yan Wang.

**Software:** Mao-Long Su, Peng-Long Wu.

**Supervision:** Yan Wang.

**Validation:** Bin Wang.

**Visualization:** Mao-Long Su.

**Writing – original draft:** Fanqi Meng.

**Writing – review & editing:** Peng-Long Wu.

## Supplementary Material


















